# Management of pseudotumor cerebri syndrome in acute promyelocytic leukemia: use of a lumboperitoneal shunt to facilitate continued ATRA therapy - a case report

**DOI:** 10.1007/s00277-026-07078-x

**Published:** 2026-05-27

**Authors:** Sara G. Creemers, Gizem Yildirim, Clemens M. F. Dirven, Nick Wlazlo

**Affiliations:** 1 Department of Hematology, Erasmus MC Cancer Institute, University Medical Center, Dr. Molewaterplein 40, Rotterdam, 3015 GD The Netherlands; 2 Department of Neurosurgery, Erasmus MC, University Medical Center, Dr. Molewaterplein 40, Rotterdam, 3015 GD The Netherlands

**Keywords:** Acute promyelocytic leukemia, All-trans retinoic acid, Pseudotumor cerebri syndrome, Lumboperitoneal shunt

## Abstract

Acute promyelocytic leukemia (APL) is associated with high early mortality, primarily due to hemorrhagic complications. Although treatment with all-trans retinoic acid (ATRA) and arsenic trioxide (ATO) has dramatically improved outcomes, ATRA may induce severe adverse effects such as pseudotumor cerebri syndrome (PTCS). We report a 19-year-old female with high-risk APL who developed recurrent PTCS during induction and consolidation therapy with ATRA. Treatment with acetazolamide and reintroduction of a lower dosages proved insufficient, and repeated lumbar punctures were contraindicated due to significant procedural anxiety and patient distress. A lumboperitoneal (LP) shunt was placed, keeping intracranial pressure stable and enabling uninterrupted ATRA administration and successful completion of consolidation therapy. To our knowledge, this is the first documented use of an LP shunt to manage refractory PTCS in an APL patient. The case highlights cerebrospinal fluid diversion as a potential strategy to sustain life-saving ATRA therapy in patients with recurrent PTCS unresponsive to conservative measures.

## Introduction

Acute promyelocytic leukemia (APL) accounts for 10–15% of all acute myeloid leukemias and has historically been among the most fatal at presentation or during induction, mainly because of catastrophic bleeding disorders [1]. The distinct hemorrhagic coagulopathy associated with acute promyelocytic leukemia is the primary contributor to early mortality, which has been reported in up to 29% of cases [2].

APL is characterized by a unique t(15;17) translocation, resulting in the presence of the PML-RARα fusion transcript. This transcript disrupts normal myeloid differentiation by inducing a transcriptional block. Treatment with all-trans retinoic acid (ATRA), a vitamin A derivative, overcomes this block by binding to the retinoic acid receptor, thereby restoring the expression of genes essential for cellular maturation [3]. Arsenic trioxide (ATO) on the other hand, targets the PML moiety, inducing apoptosis [4].

From the 1980s, the introduction of ATRA and ATO, two differentiating agents, combined with anthracycline-based chemotherapy, has been crucial for the ability of curation [5]. ATRA should be administered at the earliest suspicion of APL, even when cytogenetic or molecular confirmation is not yet known. When combined with anthracycline-based chemotherapy, ATRA has been shown to induce complete remission in the vast majority of patients, with remission rates approaching 90–100% [6, 7]. ATRA has even shown to have the ability to induce complete remission as monotherapy, but consolidation therapy is necessary to achieve sustained remission [8, 9]. More recently, chemo-free regimens combining ATRA and ATO have demonstrated excellent efficacy, achieving durable remissions in nearly all patients with low- or intermediate-risk acute promyelocytic leukemia [10] and very recently also in high risk patients [11]. As such, overall survival is excellent and relapses are very uncommon.

ATRA is usually well tolerated, but may cause potential serious complications, i.e. differentiation syndrome and pseudotumor cerebri syndrome (PTCS). PTCS is a rare complication of ATRA treatment. In a retrospective series, PTCS occurred in up to 3% of patients who received ATRA, but its true incidence is unknown because of different diagnostic criteria used [12]. The pathophysiology is thought to be similar to vitamin A toxicity, where retinoids enhance cerebrospinal fluid (CSF) production. Additionally, ATRA may disturb the normal transport system, resulting in dysfunctional absorption of CSF [13]. This results in increased CSF and intracranial pressure, with clinical symptoms including headache, nausea, papilledema and cranial nerve dysfunction. In 2013, the diagnostic criteria for PTCS were revised, consisting of; (1) papilledema, (2) a normal neurologic examination except for cranial nerve abnormalities, (3) normal CSF composition, (4) normal brain parenchyma on MRI without hydrocephalus, mass or structural lesion, and (5) elevated lumbar puncture opening pressure (> 25 cm CSF) [14]. PTCS may also be diagnosed in the absence of papilledema, but then sixth nerve palsy or specific MRI characteristics need to be present [14].

Given the pivotal role of ATRA in all phases of APL treatment, the development of strategies to mitigate the risk of PTCS or enable safe reinitiation of therapy are of critical importance. Several therapeutic drug based approaches for PTCS have been suggested in literature, including topiramate, mannitol, glycerin, analgesics, corticosteroids and acetazolamide [12, 15]. However, the limited number of reported cases hampers direct comparison of treatment efficacy and limits generalizability. Cerebrospinal fluid (CSF) shunting has been suggested as a potential intervention to alleviate PTCS-related symptoms [16], although its application in ATRA-induced PTCS within the context of APL has not been previously documented. Here, we report a case of APL complicated by ATRA-induced PCTS in which lumboperitoneal shunting enabled continuation of ATRA therapy, resulting in complete hematological remission.

## Case description

A 19-year-old female patient presented with symptoms of disseminated intravascular coagulation (DIC), anemia, thrombocytopenia, leukocytosis, and acute kidney injury. She had an elevated BMI of 34 kg/m2 and no other medical history. A diagnosis of high-risk acute promyelocytic leukemia was made, with a t(15;17)(q24;q21) translocation, suspected extramedullary disease in the kidneys based on a bilateral hypodense appearance of the kidneys on computed tomography scan (leukemic infiltration), and no central nervous system involvement. Induction therapy with ATRA 12,5mg/m^2^ and idarubicin 12mg/m^2^ on day 1 and 3 in accordance with the standard arm (B) of the HOVON 138 protocol, was initiated.

During remission induction, the patient developed multiple complications, including ATRA differentiation syndrome, severe mucositis, and subhyaloid ocular hemorrhage. On day 10, she also developed symptoms of dizziness and headache. There were no abnormalities in the neurological examination, but a lumbar puncture showed an elevated CSF opening pressure of 37 cm CSF, with normal values at CSF analysis. Both morphology and immunophenotyping revealed no evidence of APL. MRI of the brain revealed occipitoparietal ischemia, likely secondary to the initial DIC, and no other vascular or structural abnormalities. A diagnosis of pseudotumor cerebri syndrome (PTCS) was made. PTCS was managed with interruption of ATRA, acetazolamide 250 mg and dexamethasone 5 mg twice daily. ATRA was restarted at a 50% dose reduction and later full dose. Complete hematological remission was achieved, together with normalization of kidney function and the abnormal radiological findings.

During the first consolidation cycle (idarubicin, cytarabine, ATRA), signs of elevated intracranial pressure reoccurred on day 8, as suggested by complaints of nausea and headache, which were recognized by the patient from the previous episode of PTCS. A lumbar puncture technically failed due to the habitual constitution and high procedural anxiety. ATRA was temporarily discontinued. After symptomatic improvement on acetazolamide, ATRA was (again) reintroduced at 50% of the original dose, co-administered with acetazolamide. Nevertheless, PTCS recurred, this time confirmed by an opening pressure of 50 cm CSF on lumbar puncture. There was no papilledema. Symptoms improved following temporary CSF drainage by the lumbar puncture.

The pronounced procedural anxiety to lumbar punctures precluded management of recurring PTCS by repeated, pressure relieving lumbar punctures. Therefore, ATRA was permanently discontinued; at this moment 60% of the intended dose of the first consolidation cycle had been administered. Since ATRA remains the cornerstone of APL treatment, the neurosurgical team was consulted for an alternative CSF dispersing possibility. In a multidisciplinary meeting, it was decided to treat the patient with a permanent lumboperitoneal (LP) shunt. The decision to place a lumboperitoneal shunt was guided by the markedly small ventricular size observed on imaging, which rendered ventricular catheter placement potentially challenging. Furthermore, lumboperitoneal shunting avoids the risk of direct intracranial complications associated with ventriculoperitoneal shunts, such as hemorrhage and infection. Therefore, an LP shunt represents a valuable alternative in cases where elevated cerebrospinal fluid (CSF) pressure requires management through CSF drainage.

The LP shunt was placed in a surgical procedure under general anesthesia once platelet counts had adequately recovered. This allowed continuation of ATRA therapy during the subsequent consolidation cycles. To minimize the cumulative ATRA exposure, consolidation treatment was modified to another three cycles of ATO in combination with ATRA, without a maintenance phase.

Our patient completed the next consolidation cycle at full ATRA dose without neurological complications. During the third consolidation cycle, the patient developed symptoms of headache and nausea, which appeared to be caused by malfunction of the LP shunt due to dislocation of the intraperitoneal catheter into the subcutaneous tissue. Surgical revision of the shunt was performed, enabling continuation of ATRA therapy. Although a shunt dislocation of the intraspinal part of the shunt occurred at the end of the 3d consolidation cycle, the fourth and final consolidation cycle could be administered without neurological symptoms or complaints.

Nine months following completion of consolidation therapy for APL, the patient remains clinically stable and in full hematological and molecular remission.

## Discussion

To the best of our knowledge, this is the first case report demonstrating that an LP shunt may enable the continuation of ATRA treatment in APL patients with pseudotumor cerebri syndrome.

Diagnosis of PTCS can be challenging; however, based on the Naranjo Adverse Drug Reaction Probability Scale, the clinical presentation in this patient was classified as a definite adverse drug reaction (score 9), indicating a strong causal association between the administered medication and the observed adverse event [17]. Specifically the recurrence of symptoms following reintroduction of ATRA is highly suggestive of a drug-related etiology. Other predisposing factors in this case include the patient’s history of obesity, female sex, and her age [12]. It is not clearly understood why some patients are more susceptible to the development of PTCS than others. ATRA is oxidized by the cytochrome P-450 system. Although fluconazole was co-administered, its impact was deemed clinically insignificant due to its relative weak CYP3A4 inhibition. A potential relationship between fluconazole and PTCS has been suggested in a single case report; however, a causal relationship remains uncertain [18]. Besides, PTCS recurred during a shunt dislocation during the third consolidation cycle in the absence of concomitant fluconazole administration. No other relevant drug interactions were identified, and the recurrent episodes of PTCS could not be attributed to impairments in liver function or ATRA metabolism.

Therapeutic options for PTCS are variable. In a study comprising 23 cases of PTCS in APL, ATRA was withheld in 78% of patients soon after PTCS, with or without other therapeutic interventions [19]. Neurological recovery was achieved in 35% of these cases solely through cessation of ATRA. In other cases, therapeutic lumbar punctures and the use of medication like acetazolamide or mannitol, were necessary. Remarkably, only 35% of patients were rechallenged with ATRA after their neurologic condition improved. In almost all cases, like in our patient, PTCS symptoms recurred after reintroduction. Reintroducing ATRA at a lower dose alongside prophylactic administration of acetazolamide is a useful treatment approach [19]. In severe cases with impaired vision, surgical interventions may be necessary. In our patient, reintroduction with a lower dose of ATRA alongside acetazolamide did not allow for continuation of ATRA. Treatment options for PTCS when drug treatment or serial lumbar punctures are ineffective, consist of CSF shunt placements in a neurosurgical procedure. A systematic review and meta-analysis confirmed the feasibility and efficacy of CSF shunting in improving clinical symptoms, specifically papilledema and headache, in patients with idiopathic intracranial hypertension, which is a subset of PTCS [16]. This study did not include patients with APL. However, surgical shunt placement in patients with hematological malignancies and coagulation disorders, specifically the case in newly diagnosed APL, may be impossible if coagulation status cannot be corrected, due to the risk of hemorrhagic complications in the central nervous system. In this case, coagulation was already normalized by remission of the APL, after which shunt placement could safely be performed.

The two most widely used CSF shunting procedures are ventriculoperitoneal (VP) and LP shunting, with the VP shunt associated with a lower risk of revision but potentially more severe complications. Because of the very small sized ventricles, an LP shunt was deemed the safer alternative in our patient. LP shunts cause greater risk of CSF overdrainage, resulting in position dependent headache. We informed the patient about this possible risk that we deemed to be higher because she likely had normal CSF pressure values in between her treatment cycles.

After careful deliberation with the patient and considering the potential risks of not placing a shunt, we decided to proceed. She did not, however, develop any symptoms of CSF overdrainage, even though the shunt was implanted without a valve or anti-siphon mechanism. Additionally, no infectious complications occurred due to the LP drain. Notably, PTCS recurred after the first shunt dislocation, while ATRA therapy could be resumed without further CSF diversion after the second dislocation. Theoretically, this may be explained by residual effects from the previous LP drain, which could have offered a partial protective effect, allowing completion of the final two weeks of ATRA. Although no intrathecal component of the shunt remained at that time, it is plausible that a small, persistent defect at the former insertion site facilitated a low-volume CSF leakage, thereby helping to maintain tolerable intracranial pressure throughout this interval.

In conclusion, this case highlights pseudotumor cerebri syndrome as a rare but challenging complication of ATRA therapy in APL, requiring a multidisciplinary approach for continued administration of life-saving therapy. In patients with APL and repeated PTCS with contraindications to frequent lumbar punctures, early consideration of CSF diversion techniques such as lumboperitoneal shunting may facilitate uninterrupted curative treatment.

## Data Availability

No datasets were generated or analysed during the current study.
